# Establishment of a conditional TALEN system using the translational enhancer dMac3 and an inducible promoter activated by glucocorticoid treatment to increase the frequency of targeted mutagenesis in plants

**DOI:** 10.1371/journal.pone.0208959

**Published:** 2018-12-26

**Authors:** Hitomi Onodera, Saeko Shingu, Mariko Ohnuma, Takaaki Horie, Miho Kihira, Hiroaki Kusano, Hiroshi Teramura, Hiroaki Shimada

**Affiliations:** Department of Biological Science and Technology, Tokyo University of Science, Katsushika, Tokyo, Japan; RIKEN Biomass Engineering Program, JAPAN

## Abstract

Transcription activator-like effector nuclease (TALEN) is an artificial nuclease that causes DNA cleavage at the target site and induces few off-target reactions because of its high sequence specificity. Powerful and variable tools using TALENs can be used in practical applications and may facilitate the molecular breeding of many plant species. We have developed a convenient construction system for a plant TALEN vector named the Emerald Gateway TALEN system. In this study, we added new properties to this system, which led to an increase in the efficiency of targeted mutagenesis. Rice dMac3 is a translational enhancer that highly increases the efficiency of translation of the downstream ORF. We inserted dMac3 into the 5' untranslated region of the TALEN gene. In the cultured rice cells to which the TALEN gene was introduced, the frequency of targeted mutagenesis was highly increased compared with those altered using the conventional system. Next, the promoter for the TALEN gene was replaced with iPromoter, and its expression was stringently controlled by a GVG transcription factor that was activated in the presence of glucocorticoid. This conditional expression system worked effectively and led to a higher frequency of targeted mutagenesis than that by the constitutive expression system, while no mutagenesis was detected without glucocorticoid treatment. These results suggest that our system can be applied to genome editing to create the desired mutation.

## Introduction

Genome-editing technologies based on designed artificial nucleases, such as zinc finger nuclease, TALEN, and clustered regularly interspaced short palindromic repeat (CRISPR)/Cas9, have emerged in the recent decade and currently enable the precise and efficient genetic modification of target genes of various cells and organisms [1–2]. To obtain the desired mutant, a powerful and user-friendly system that is applicable for many species is needed.

In the case of CRISPR/Cas9, the CRISPR RNA directs Cas9 to introduce double-stranded breaks in target DNA [3]. The targeting specificity of Cas9 is regulated by the 20-nt guiding sequence of a single guide RNA and the presence of a protospacer-adjacent motif (PAM) adjacent to the target sequence in the genome [4]. However, the high frequency of off-target activity creates mutations at sites other than the intended on-target site [5].

On the other hand, off-target problems are low in the TALEN system. TALEN is composed of transcription activator-like effectors (TALEs) fused to the catalytic domains of the *FokI* endonuclease and causes double-strand breaks only upon dimerization of the *FokI* domains [6]. The methods for the targeted mutagenesis of plant genomes using TALENs have been reported [7,8]. In general, TALEN consists of 18 repetitions of 34 amino acids in which the 12th and 13th residues determine DNA binding specificity and are called the "repeatable variable di-residues" (RVD) [9,10]. The TALEN pair must bind to opposite strands split by a "spacer" consisting of 14 to 20 bases at the target site. For *FokI* to be active, it must form a dimer, so this offset design is required [11]. The degeneracy of the RVD-DNA binding code may be observed [12]. However, TALEN rarely induces an off-target reaction because of DNA cleavage at the target site with high sequence specificity [13,14].

The 5' untranslated region (5'UTR) of some mRNAs is known to function as an enhancer of translation, largely increasing the production of the protein encoded by the downstream ORF [15]. We have found long 5'UTRs that show strong translational enhancer activity in the rice genes *OsMac1*, *OsMac2*, and *OsMac3*. They lead to 10-fold or higher efficiency of translation of the downstream ORFs. Among them, the 5'UTR of *OsMac3* mRNA was shown to be a strong translational enhancer that significantly promotes the translational efficiency of the downstream ORF [16].

We have established a simple TALEN construction system, named the Emerald Gateway TALEN system, which employs Gateway-assisted plasmid construction to produce the desired TALEN genes [17]. In this study, we attempted to increase the efficiency of targeted mutagenesis by applying the enhancer activity of the 5'UTR of *OsMac3* to our TALEN construction system.

For plant genome editing, artificial nucleases ubiquitously expressed in plant cells are generally used [18]. However, it has been shown that the constitutive CaMV-35S promoter causes a mosaic effect with a high mutation rate [19]. Therefore, to avoid such a harmful event, it is desired that any activities of the artificial nucleases are diminished after finishing the genome-editing event.

For this purpose, we attempted to construct an artificial nuclease gene that showed conditional expression under an inducible promoter. Aoyama and Chua have reported an inducible promoter system consisting of two components containing the gene for the transcription factor GVG and the specialized promoter that responds to the activated GVG protein [20]. GVG is a chimeric transcription factor consisting of the DNA-binding domain of the yeast transcription factor GAL4, the trans-activating domain of the herpes viral protein VP16, and the receptor domain of the rat glucocorticoid receptor (GR), which is activated by the addition of glucocorticoid [20]. In this study, we applied this system to our TALEN construction system. We expected that the resultant system would be controlled stringently by the addition of glucocorticoid.

Here, we report the construction of novel TALEN vector systems that enhance the efficiency of target mutagenesis, and we show the results of an evaluation of the mutagenesis using TALENs with the chemically controlled promoter.

## Materials and methods

### Plant materials and culture conditions

For the rice cultured cells, *Oryza sativa* L. cv. Nipponbare was used. Cells were cultured on N6D medium [21] at 28°C under continuous light conditions in a growth chamber.

### Plasmid construction

**Fig 1:**
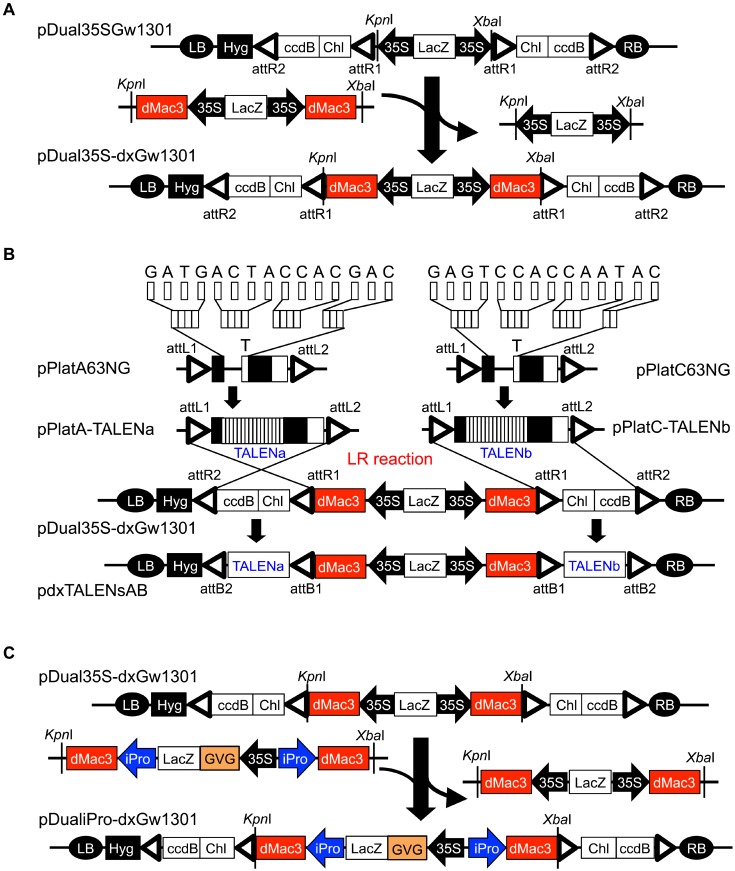
Outline of the Emerald Gateway dx-TALEN system. (A) Construction of pDual35S-dxGw1301. (B) Schematic representation of the TALEN construction procedure using the Emerald Gateway dx-TALEN system. (C) Construction of pDualiPro-dxGw1301. pDual35S-dxGw1301 and pDualiPro-dxGw1301 are destination vectors and pPlatA63NG and pPlatC63NG are entry vectors that used for the Emerald Gateway TALEN system and its improved systems [17]. dMac3: dMac3 translational enhancer, 35S: CaMV 35S promoter, ccdB: ccdB toxin gene, Chl: Chloramphenicol resistance gene, Amp: Ampicillin resistance gene, Hyg: Hygromycin resistance gene, LacZ: LacZ gene, attL1, attR1, attL2, attR2, attB1, and attB2: attL1, attR1, attL2, attR2, attB1, and attB2 sequences for the Gateway reaction, respectively, *Esp*3I, *Kpn*I, and *Xba*I: cleavage sites of *Esp*3I, *Kpn*I, and *Xba*I, respectively, GVG: the gene for GVG transcription factor, 35S::GUS: fusion construct of the CaMV 35S promoter and GUS gene, iPro: iPromoter, RB: T-DNA right border, LB: T-DNA left border.

Destination vectors, pDual35S-dxGw1301 and pDualiPro-dxGw1301, were constructed based on pDual35SGw1301, a destination vector in the Emerald Gateway TALEN system [17]. pDual35S-dxGw1301 is a destination vector employing the dMac3 translational enhancer preceding the entry site for the TALEN genes. A fragment corresponding to two regions of dMac3 with the 35S promoter was chemically synthesized with the LacZ gene included in between the two regions. This fragment was replaced with the region of the component consisting of two 35S promoter regions of pDual35SGw1301, and pDual35S-dxGw1301 was generated (Fig 1 and Fig A in S1 File).

pDualiPro-dxGw1301 contains the iPromoter, which is activated by the transcription factor GVG in the presence of glucocorticoid [20]. A fragment consisting of dMac3 with iPromoter was chemically synthesized. The nucleotide sequence of the iPromoter was taken from the previously reported one [20], in which three nucleotides were substituted to remove the two *Xba*I sites and *Kpn*I site (Fig A in S1 File). A fragment for GVG was PCR-amplified from the plasmid pTA7002 [20] using the primers 5'–CCTGTTCTCGAGAGTCCCCAGATTAGCCTTTTCAA–3' and 5'–CGCGCCACTAGTAGTGTTTTACTCCTCATATT–3'. These fragments were connected in the following order: the fragment for dMac3 with iPromoter, the fragment for GVG, and the fragment for iPromoter followed by dMac3, to generate a fragment consisting of two sets of dMac3 with iPromoter, between which the region for GVG was contained. The resultant fragment was replaced with the component containing dMac3 with the 35S promoter of pDual35S-dxGw1301, and pDualiPro-dxGw1301 was generated (Fig 1 and Fig A in S1 File).

TALEN genes used in this study were constructed as follows: The target sites for the TALENs used in this study were designed by TAL Effector-Nucleotide Targeter 2.0 software (https://tale-nt.cac.cornell.edu/) [22]. The sequence 5'–TGATGACTACCACGACTatcttggtacctttgAGTATTGGTGGACTCA–3' in the rice *Os01g0833500* gene (acc. No. AK073320) was used as the target of TALENs. In this sequence, the regions shown in capital letters were adopted as the recognition sequences for TALENs. Components of TALENs were constructed using pPlat plasmids using the Emerald Gateway TALEN system [17]. The resultant TALEN genes were assembled into pDual35SGw1301, pDual35S-dxGw1301, and pDualiPro-dxGw1301 by Gateway LR Clonase II (Invitrogen, Carlsbad, USA). The generated binary vectors were used for targeted mutagenesis.

### Plant transformation and glucocorticoid treatment conditions

Rice callus cells were transformed using the *Agrobacterium*-mediated procedure with the *A*. *tumefaciens* EHA105 strain [23] according to Toki et al. [24]. Transformed cells were selected by acquired hygromycin resistance on an agar plate of N6D medium supplemented with 50 mg L^-1^ hygromycin B (Wako Pure Chemicals, Osaka, Japan) and cultured for four weeks. An inducible promoter controlled by the transcription factor GVG was activated by the addition of glucocorticoid (Sigma-Aldrich, St. Louis, USA). A 30 mM glucocorticoid solution in ethanol was applied to the culture medium to reach a concentration of 60 μM. Callus cells were cultured on the plate containing glucocorticoid after they were infected by *Agrobacterium* harboring TALEN genes and maintained for three days. Then, they were placed onto N6D plates supplemented with 50 mg L^−1^ hygromycin B and 60 μM glucocorticoid to select transgenic lines for five days.

### CAPS analysis

Genomic DNA was prepared using a DNA preparation kit and QIAshredder with RLT lysis buffer (QIAGEN, Hilden, Germany). The 0.7-kb region in the *Os01g0833500* gene that contained the target sites for the TALENs was PCR-amplified using the primers 5'–GCCCTGATTTACCATGATTC–3' and 5'–GTCAAGAGGGTGATCTAAG–3'. The amplified fragment was digested with *Kpn*I. The wild-type gene has a *Kpn*I site in the corresponding region. Therefore, a mutant allele was called when the amplified fragment was no longer digested by *Kpn*I. The number of mutant alleles was estimated by the ratio of undigested fragments to digested fragments.

### Sequence analysis

The fragment corresponding to the same region used for CAPS analysis that was amplified using the primers 5'–CCTGTTGGGCCCATGAGTACAGGGAGTTCTACATTGC–3' and 5'–ATGGCCGAATTCGCAGTCCTCCCAAGCCAAGTTTG–3' was treated with *Kpn*I. Then, the resultant fragments were inserted into the pDONR207 vector (Invitrogen, Carlsbad, USA) after digestion with *Apa*I and *Eco*RI. The plasmids obtained were used for nucleotide sequence analysis.

### RT-PCR

Total RNA was prepared from rice callus using an RNeasy Plant Mini kit (QIAGEN, Hilden, Germany). First-strand cDNA was synthesized from 3 μg of total RNA using the ReverTra-Ace cDNA synthesis kit (Toyobo, Osaka, Japan) with an oligo-dT (20) primer. The resultant cDNA was used for the detection of transcripts corresponding to the region of the *FokI* gene in the TALEN genes. RT-PCR was performed using the primers 5'–CTCGACTCAAGACAGAATCC–3' and 5'–GACTTCTTCCAGTGTCAG–3' with a high-fidelity KOD FX neo DNA polymerase (Toyobo, Osaka, Japan). The transcript for *actin1* (acc. No. AB047313) was analyzed as the control and used for normalization of the data; the transcript was amplified using the primer set 5'–AGCTTCCTGATGGACAGGTT–3' and 5'–GTCCGAAGAATTAGAAGCATTTCC–3'.

## Results

### Construction of the TALEN vector harboring a translational enhancer and the promoter stringently controlled by the addition of a chemical reagent

We have developed a vector construction system for plant genome editing, named the Emerald Gateway TALEN system, which was utilized to construct a TALEN vector using Gateway cloning technology [17]. In this study, we improved this system to enrich the genome cleaving activity. Recently, we reported that the 5' untranslated region of OsMac3 mRNA significantly enhanced the translation efficiency of the downstream ORF [16]. We also found that a portion of the 5' untranslated region of OsMac3 mRNA consisting of 161 nucleotides, named dMac3, showed sufficient activity as a translational enhancer.

To insert dMac3 into the Emerald Gateway TALEN system, the fragment corresponding to dMac3 was inserted into the destination vector of the Emerald Gateway TALEN system, pDual35SGw1301 (Fig 1A). The resultant plasmid, pDual35S-dxGw1301, was utilized for the construction of the genes for a pair of TALENs, each of which was translated from the mRNA containing dMac3 preceding the protein-coding region. This system was designated the Emerald Gateway dx-TALEN system (Fig 1B). In this system, it was expected that a large number of the TALEN molecules will be produced from the TALEN genes driven by the 35S promoter.

Next, we aimed to change the promoter that was stringently expressed in the presence of a chemical reagent into pDual35S-dxGw1301. We inserted a promoter, named iPromoter, which is activated by the transcription factor GVG in the presence of glucocorticoid [20]. This promoter is composed of a glucocorticoid receptor (6xGAL4 UAS) and a transcriptional activator [20]. A fragment corresponding to this promoter and the transcription factor GVG gene was replaced with the promoter region of pDual35S-dxGw1301 to construct a destination vector, pDualiPro-dxGw1301 (Fig 1C). This system was designated as Emerald Gateway dx-TALEN premium system.

**Fig 2:**
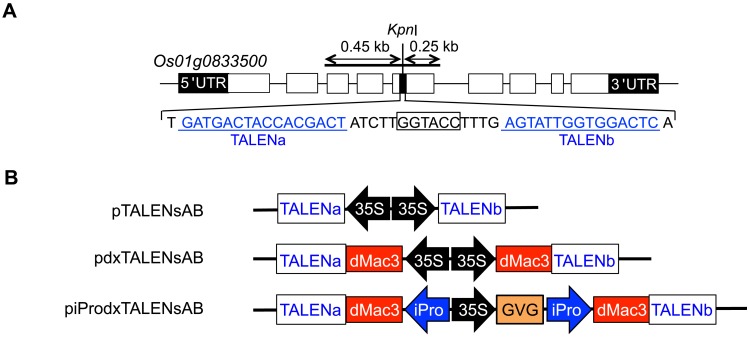
Structure of the TALEN genes. (A) Target regions for the TALENs used in this study. The nucleotide sequence of the target region is shown below the structure of the *Oryza sativa 01g0833500* gene. Exons are shown in boxes. Target sequences for the TALENs are underlined. The *Kpn*I site is boxed. The region for the PCR-amplified fragment for CAPS analysis is shown above the structure. (B) Structure of the TALEN genes based on Emerald Gateway TALEN (pTALENsAB), dx-TALEN (pdxTALENsAB) and dx-TALEN premium (piProTALENsAB). Elements on this figure are the same as those in Fig 1.

We constructed TALEN genes that targeted a rice protease gene, *Os01g0833500* (Fig 2A). Using each TALEN construction system, the Emerald Gateway TALEN system, Emerald Gateway dx-TALEN system, and Emerald Gateway dx-TALEN premium system, we obtained specific pairs of TALEN genes, which were pTALENsAB, pdxTALENsAB, and piProdxTALENsAB, respectively (Fig 2B).

### Evaluation of the efficiency of targeted mutation using the dMac3 translational enhancer

**Fig 3:**
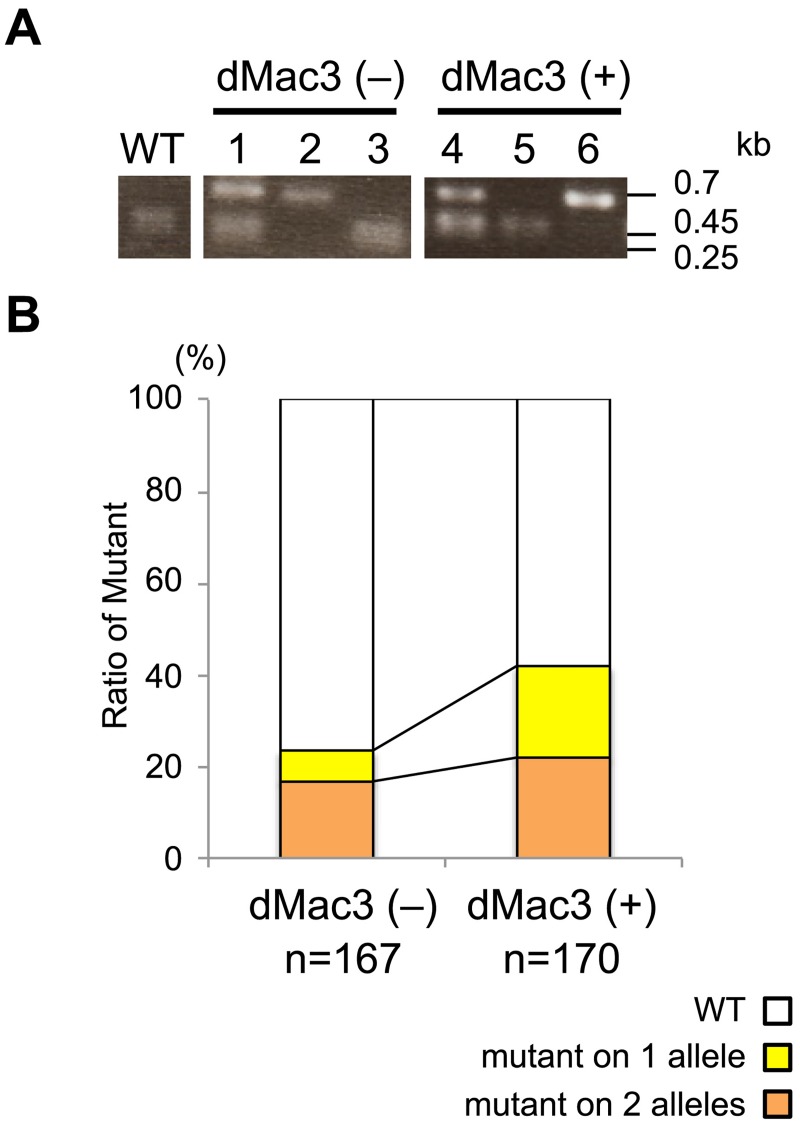
Efficiency of targeted mutagenesis using pDual35S-dxGw1301. (A) Detection of mutant alleles by CAPS analysis. A fragment containing the target site was PCR amplified and digested with *Kpn*I. WT: wild-type, dMac3(–): transformant callus containing pTALENsAB without dMac3, dMac3(+): transformant callus containing pdxTALENsAB with dMac3. Lanes 1 to 3 indicate the results of CAPS analysis, which show one allele, two alleles, and no allele mutation, respectively. WT shows the wild-type allele. Sizes of fragments are shown on the right side. (B) Graphical representation of the mutation rate in the transformants harboring pTALENsAB and pdxTALENsAB. Mutant alleles are determined by CAPS analysis.

To evaluate the effect of the translational enhancer dMac3 on the induction of targeted mutagenesis, pTALENsAB and pdxTALENsAB were introduced into rice callus, and cultured for four weeks. Significant difference was not detected between the amount of the transcript for the TALEN gene in the transformed callus containing pTALENsAB and pdxTALENsAB (Fig B in S1 File). A *Kpn*I restriction site was contained in the target region, and we detected the mutant alleles in the transformant callus cells by CAPS analysis (Fig 3A). When the 0.7-kb fragment was located around the target sequence, it was cleaved into 0.25-kb and 0.45-kb fragments by *Kpn*I digestion in the wild-type cells. The frequency of mutations generated was determined using the ratio of *Kpn*I-digested/undigested PCR-amplified fragments containing the target sites (Fig B in S1 File).

We determined the ratio of targeted mutagenesis that occurred depending on the dMac3 enhancer sequence. Numbers of mutant alleles in the transformants significantly increased when pdxTALENsAB was used. In this case, mutant alleles were detected in 71 of 170 (41.8%) transformants containing dMac3, whereas the mutant alleles were detected in 39 of 167 (23.4%) transformants containing no enhancer sequence (Fig 3B; Table A in S1 File). Mutations in multiple alleles occurred in 37 (21.8%) transformants containing dMac3 but in only 28 (16.8%) transformants containing no enhancer sequence (Fig 3B; Table A in S1 File). These results suggested that the number of mutations was greatly increased in the transformants harboring the dMac3 vector.

### Induced targeted mutagenesis using iPromoter

**Fig 4:**
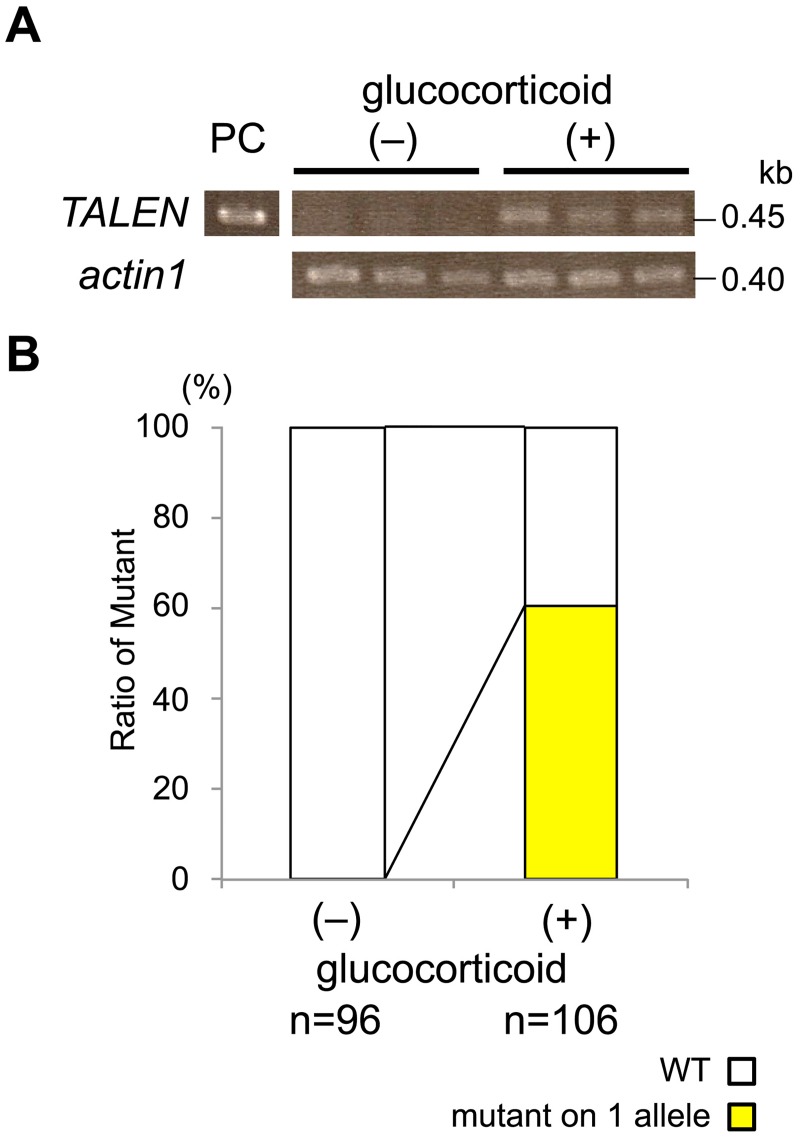
Efficiency of targeted mutagenesis using pDualiPro-dxGw1301. (A) Detection of the TALEN gene transcript by RT-PCR. A transcript corresponding to the region containing the target site was amplified. As a control, the transcript of the rice actin gene (*actin1*) was analyzed. (–) and (+) indicate the transformants harboring piProTALENsAB treated with/without glucocorticoid in the culture medium. (B) Graphical representation of the mutation rate in the transformants harboring piProdxTALENsAB treated with/without glucocorticoid. Mutant alleles were determined by CAPS analysis. (+) and (–) indicate the transformants treated with/without glucocorticoid, respectively. WT indicates the wild-type allele.

Using piProdxTALENsAB, rice transformant calli were generated. They were cultivated on medium in the presence or absence of glucocorticoid, and evaluated for targeted mutagenesis. To induce the TALEN gene expression, glucocorticoid was added in the cultured medium both for infection of *Agrobacterium* and selection of transformant cells. RT-PCR was carried out to determine the expression of the introduced TALEN genes in the hygromycin-resistant callus that were cultured for two days. As shown in Fig 4A, a sufficient amount of the transcript for TALEN genes was detected in the cells on the medium containing glucocorticoid, suggesting that the introduced TALEN genes were stringently expressed in the presence of glucocorticoid.

Numbers of mutant alleles in the transformants that were cultured for five days were determined by CAPS analysis. In this case, mutant alleles were found in 64 of 106 (60.4%) transformants treated with glucocorticoid, whereas no mutations were detected in 96 (0%) transformants that were not treated with glucocorticoid (Fig 4B; Table B in S1 File). These results indicated that induced targeted mutagenesis occurred specifically by the TALEN gene with iPromoter. However, none of multiple alleles was detected in these transformants (Fig 4B; Fig B in S1 File).

### Sequence analysis of the target site in the mutant alleles

**Fig 5:**
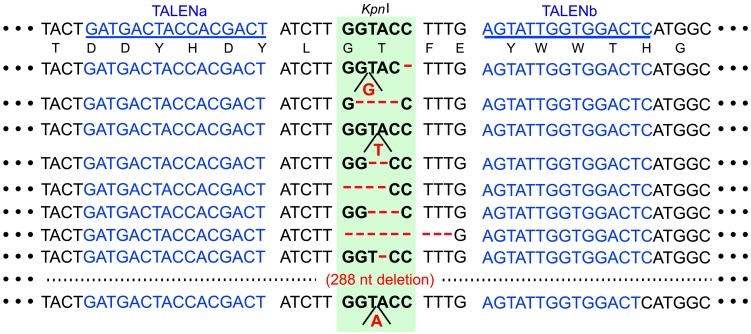
Nucleotide sequences around the target site of the representative mutants. The corresponding wild-type nucleotide sequence (WT) with deduced amino acid sequence is shown in the upper portion of the figure. The nucleotide sequences of TALENa and TALENb are underlined. The region corresponding to the *Kpn*I site is shaded. Numbers with the prefix # indicate the individual transformants. Gaps indicate the nucleotide deletion in the mutants, and nucleotide insertions are shown below.

We determined the nucleotide sequences of the target region of the TALENs in the representative 10 mutant calli. The corresponding region was PCR-amplified from the calli containing piProdxTALENsAB and then digested by *Kpn*I. The resultant DNA was subcloned, and their nucleotide sequences were determined. In addition to the wild-type sequence, we detected different types of mutations and polymorphic DNA sequences around the target region of the mutants in this region. Among them, 6 mutants contained small numbers (1–9) of nucleotide deletions. Two mutants contained one nucleotide insertion, and one mutant contained one nucleotide insertion and one nucleotide deletion. One mutant contained 288 nucleotide deletion (Fig 5).

## Discussion

Powerful and variable tools using TALENs are desired for plant genome editing because they can be used for practical applications in the molecular breeding of many plant species, which may supply a new method for the production of foods, medicals, and some other materials. Here, we propose a new strategy enabling high-efficiency targeted mutagenesis.

dMac3 shows translation enhancer activity that efficiently increases the amount of protein generated from the downstream ORF [16]. Recently, we reported that dMac3 employed in conjunction with the Cas9 gene of a CRISPR/Cas9 vector system may allow increases in the frequency of targeted mutagenesis in potato cells [25]. It is expected that TALEN genes employing dMac3 would produce a large number of the TALEN molecules in the cells when dMac3 was set preceding the ORFs for TALENs. We expected that cleavage of the target site would occur very frequently in this cell, and consequently, the efficiency of targeted mutagenesis would increase. In this study, we constructed two types of conventional TALEN construction systems, the Emerald Gateway dx-TALEN system and Emerald Gateway dx-TALEN premium system, which enabled the creation of a pair of TALEN genes corresponding to an appropriate region of the target gene (Fig 1). Using these systems, the generation of a large number of the TALEN molecules is expected in the cells with ubiquitous and inducible expression.

Using Emerald Gateway dx-TALEN system, we determined the effect of dMac3 on genome editing. We constructed TALEN genes that targeted the sequences in the *Os01g0833500* gene where there was a *Kpn*I cleavage site. We analyzed targeted mutation at this site by CAPS analysis as the disappearance of *Kpn*I cleavage and determined the efficiency of mutagenesis by the presence or absence of dMac3. The frequency of mutagenesis was greatly increased when the TALENs with dMac3 were used (Fig 3). This suggests that dMac3 is an effective tool for the induced mutation by TALENs.

To reduce the potential toxic effects of genome editing reagents, the activity of these reagents needs to be controlled by incorporating regulatory switches that can reduce off-target activities and/or allow for these reagents to be turned on or off [26]. We expected that the artificial nucleases could work in the desired organs with specificity in a temporal manner. iPromoter is a strong promoter stringently controlled by the GVG transcription factor, which is activated in the presence of glucocorticoid [20]. To make TALENs controlled by the inducible promoter, we created a TALEN construction system employing the dMac3 translational enhancer with iPromoter (Fig 1C).

TALENs corresponding to the *Os01g0833500* gene were constructed, and the efficiency of mutation in the cultured cells was analyzed. Mutations were specifically and frequently detected in the glucocorticoid-treated cells (Fig 4). These mutant cells contained nucleotide deletions, insertions, and rearrangements in the region targeted by the TALENs (Fig 5). These results suggested that this system allowed targeted mutagenesis to occur dependent on induction by glucocorticoid treatment. However, no two-allele mutation was found in the cells treated by the TALENs with iPromoter (Fig 4), whereas a large number of two-allele mutants were detected in the callus by the TALENs with dMac3 (Fig 3). In this study, glucocorticoid was applied to the transformant callus for 5 days. We presume that the period of glucocorticoid treatment was too short to induce the targeted mutation in both of two alleles. The fact that mutation occurred in more than 60% calli implies that this system effectively works for the targeted mutagenesis. Our system may facilitate the creation of appropriate TALEN genes that target arbitrary sequences and may be a powerful tool for genome editing.

## Conclusion

TALEN is an artificial nuclease that causes DNA cleavage at the target site and induces few off-target reactions. We added new properties to the TALEN construction system. We inserted dMac3, a translational enhancer highly increasing the efficiency of translation of the downstream ORF, into the 5' untranslated region of the TALEN gene. In the cultured rice cells to which the TALEN gene was introduced, the frequency of targeted mutagenesis was highly increased. Next, the promoter for the TALEN gene was replaced with an inducible promoter, iPromoter. Its expression was stringently controlled by a GVG transcription factor that was activated in the presence of glucocorticoid. This conditional expression system worked effectively and led to a higher frequency of targeted mutagenesis, while no mutagenesis was detected without glucocorticoid treatment. These results suggest that our system can be applied to genome editing to create the desired mutation.

## Supporting information

S1 File Table A. Ratio of mutation in the transformant callus harboring pdxTALENs. Table B. Ratio of mutation in the transformant callus harboring piProdxTALENs. Fig A. Details of pDual35S-dxGw1301 and pDualiPro-dxGw1301. (A) Structure of the core region of pDual35S-dxGw1301 and pDualiPro-dxGw1301. 35S: CaMV 35S promoter, iPro: iPromoter, dMac3: dMac3 translational enhancer, ccdB: ccdB toxin gene, Chl: Chloramphenicol resistant gene, attR1 and attR2: attR1 and attR2 sequences for Gateway reaction. (B) Nucleotide sequence of the core region of pDual35S-dxGw1301. Regions for 35S promoter, dMac3, and the following attR1 sequences are shown. dMac3 region is boxed. attR1 sequence is underlined. The initiation codon is indicated on the figure. (C) Nucleotide sequence of the core region of pDualiPro-dxGw1301. Regions for iPromoter, dMac3, and the following attR1 sequences are shown. In the iPromoter, substituted nucleotides from the previously reported sequence [20] are indicated by red colored letters (G to A, T to A, and C to T, respectviely). dMac3 region is boxed. attR1 sequence is underlined. The initiation codon is indicated on the figure. Fig B. Detection of targeted mutation. (A) CAPS analysis of the transformant cali harboring TALEN genes with/without dMac3. Polymorphic DNAs around the target region were detected in the transformants. dMac3 (–) and dMac3 (+) indicate the callus containing pTALENsAB and pdxTALENsAB, respectively. Numbers show the individual callus. Sizes of the fragments are shown on the right. (B) Detection of the transcripts of TALEN genes. Transcripts for TALEN genes and actin1 are detected by semi-quantitative RT-PCR. dMac3 (–) and dMac3 (+) indicate the callus containing pTALENsAB and pdxTALENsAB, respectively. Numbers on the lanes indicate the individual callus. PC indicates the fragment amplified from the control plasmid. M indicates the size marker. Sizes of the fragments are shown on the right. (C) CAPS analysis of the transformant cali harboring piProTALENsAB with/without treatment of glucocorticoid. Polymorphic DNAs around the target region were detected in the transformants. Upper panel (glucocorticoid (+)) show the results of the CAPS analysis of the transformant callus treated with glucocorticoid, and lower panel (glucocorticoid (–)) show those without glucocorticoid treatment. Numbers show the individual callus. Sizes of the fragments are shown on the right.
(PDF)

## Abbreviations

CAPS: cleaved amplified polymorphic sequence
ORF: open reading frame
RT-PCR: reverse transcriptase mediated PCR
