# Supplementary material for: Establishment of a conditional TALEN system using the translational enhancer dMac3 and an inducible promoter activated by glucocorticoid treatment to increase the frequency of targeted mutagenesis in plants
Source: PLoS One. 2018 Dec 26;13(12):e0208959. doi: 10.1371/journal.pone.0208959 (PMC6306166; doi:10.1371/journal.pone.0208959)
Supplement: S1 File — Table A. Ratio of mutation in the transformant callus harboring pdxTALENs. Table B. Ratio of mutation in the transformant callus harboring piProdxTALENs. Fig A. Details of pDual35S-dxGw1301 and pDualiPro-dxGw1301. (A) Structure of the core region of pDual35S-dxGw1301 and pDualiPro-dxGw1301. 35S: CaMV 35S promoter, iPro: iPromoter, dMac3: dMac3 translational enhancer, ccdB: ccdB toxin gene, Chl: Chloramphenicol resistant gene, attR1 and attR2: attR1 and attR2 sequences for Gateway reaction. (B) Nucleotide sequence of the core region of pDual35S-dxGw1301. Regions for 35S promoter, dMac3, and the following attR1 sequences are shown. dMac3 region is boxed. attR1 sequence is underlined. The initiation codon is indicated on the figure. (C) Nucleotide sequence of the core region of pDualiPro-dxGw1301. Regions for iPromoter, dMac3, and the following attR1 sequences are shown. In the iPromoter, substituted nucleotides from the previously reported sequence [20] are indicated by red colored letters (G to A, T to A, and C to T, respectviely). dMac3 region is boxed. attR1 sequence is underlined. The initiation codon is indicated on the figure. Fig B. Detection of targeted mutation. (A) CAPS analysis of the transformant cali harboring TALEN genes with/without dMac3. Polymorphic DNAs around the target region were detected in the transformants. dMac3 (–) and dMac3 (+) indicate the callus containing pTALENsAB and pdxTALENsAB, respectively. Numbers show the individual callus. Sizes of the fragments are shown on the right. (B) Detection of the transcripts of TALEN genes. Transcripts for TALEN genes and actin1 are detected by semi-quantitative RT-PCR. dMac3 (–) and dMac3 (+) indicate the callus containing pTALENsAB and pdxTALENsAB, respectively. Numbers on the lanes indicate the individual callus. PC indicates the fragment amplified from the control plasmid. M indicates the size marker. Sizes of the fragments are shown on the right. (C) CAPS analysis of the transformant cali harboring [file pone.0208959.s001.pdf]

Table A. Ratio of mutation in the transformant callus harboring pdxTALENs.

dMac3	mutant on 1 allele	mutant on 2 alleles	WT	Total
–	11 (6.6%)	28 (16.8%)	128	167
+	34 (20.0%)	37 (21.8%)	99	170

Table B. Ratio of mutation in the transformant callus harboring piProdxTALENs.

glucocorticoid	mutant on 1 allele	mutant on 2 alleles	WT	total
–	0	0	96	96
+	64 (60.4%)	0	42	106

(A)

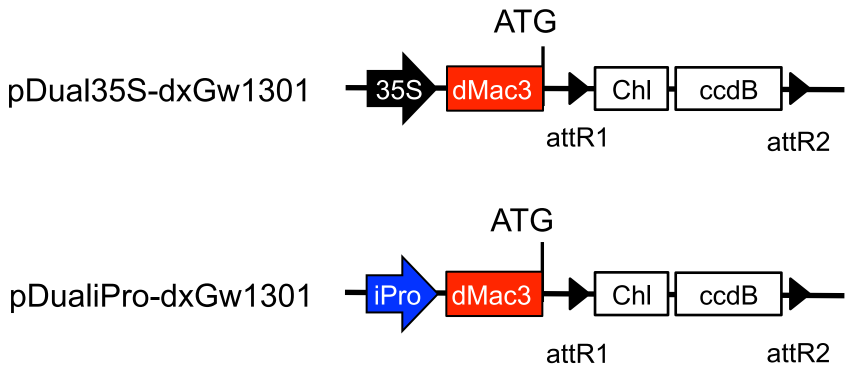

(B)

35S promoter

CATGGAGTCAAAGATTCAAATAGAGGACCTAACAGAACTCG
CCGTAAAGACTGGCGAACAGTTCATACAGAGTCTCTTACGA
CTCAATGACAAGAAGAAAATCTTCGTCAACATGGTGGAGCA
CGACACACTTGTCTACTCCAAAAATATCAAAGATACAGTCT
CAGAAGACCAAAGGGCAATTGAGACTTTTCAACAAAGGGTA
ATATCCGGAAACCTCCTCGGATTCCATTGCCCAGCTATCTG
TCACTTTATTGTGAAGATAGTGGAAAAGGAAGGTGGCTCCT
ACAAATGCCATCATTGCGATAAAGGAAAGGCCATCGTTGAA
GATGCCTCTGCCGACAGTGGTCCCAAAGATGGACCCCCACC
CACGAGGAGCATCGTGGAAAAAGAAGACGTTCCAACCACGT
CTTCAAAGCAAGTGGATTGATGTGATATCTCCACTGACGTA
AGGGATGACGCACAATCCCACTATCCTTCGCAAGACCCTTC
CTCTATATAAGGAAGTTCATTTCATTTGGAGAGAACACGGG
GGACTCTTGACCTCTAGTAAGACTAAAGAGAGCTTTTTCAT

dMac3

ACCAAAGAAGTACAACAAAAGATTTGCTCCTCATTTTCTGA
ATCCTGGGACTCTCTAGCCTGTAGAAGAAGAAAGGCAGGAA
TTTCAGCTCAAGAGAACAGATCACAATATTTACCCACGGCA
CTGTCTCGCAAT**ATG**AGATCTGGTCTAGAGTTATCAACAAG

attR1

TTTGTACAAAAAAGCTGAACGAGAAACGTAAAATGATATAA
ATATCAATATATTAAATTAGATTTTGCATAAAAAACAGACT
ACATAATACTGTAAAACACAACATATCCAGTCACTATGCAT

initiation codon

(C)

iPromoter

CATGCCGGTCGACTCTAAAGGATCCGGGTGACAGCCCTCCG
ACGGGTGACAGCCCTCCGACGGGTGACAGCCCTCCGAATTC
AAGAGGATCCGGGTGACAGCCCTCCGACGGGTGACAGCCCT
CCGACGGGTGACAGCCCTCCGAATTCGAGCTCGGTATCCGG
GGATCTGTCGACCTCGATCGAGATCTTCGCAAGACCCTTCC
TCTATATAAGGAAGTTCATTTCATTTGGAGAGGACACGCTG
AAGCTAGTCGACTCTAGCATTTAAATAAGACTAAAGAGAGC

dMac3

TTTTTCATACCAAAGAAGTACAACAAAAGATTTGCTCCTCA
TTTTCTGAATCCTGGGACTCTCTAGCCTGTAGAAGAAGAAA
GGCAGGAATTTCAGCTCAAGAGAACAGATCACAATATTTAC
CCACGGCACTGTCTCGCAAT**ATG**TGCGGACGTATTCTAGAG

attR1

TTATCAACAAGTTTGTACAAAAAAGCTGAACGAGAAACGTA
AAATGATATAAATATCAATATATTAAATTAGATTTTGCATA
AAAAACAGACTACATAATACTGTAAAACACAACATATCCAG
TCACTATGCAT

initiation codon

Fig A. Details of pDual35S-dxGw1301 and pDualiPro-dxGw1301.

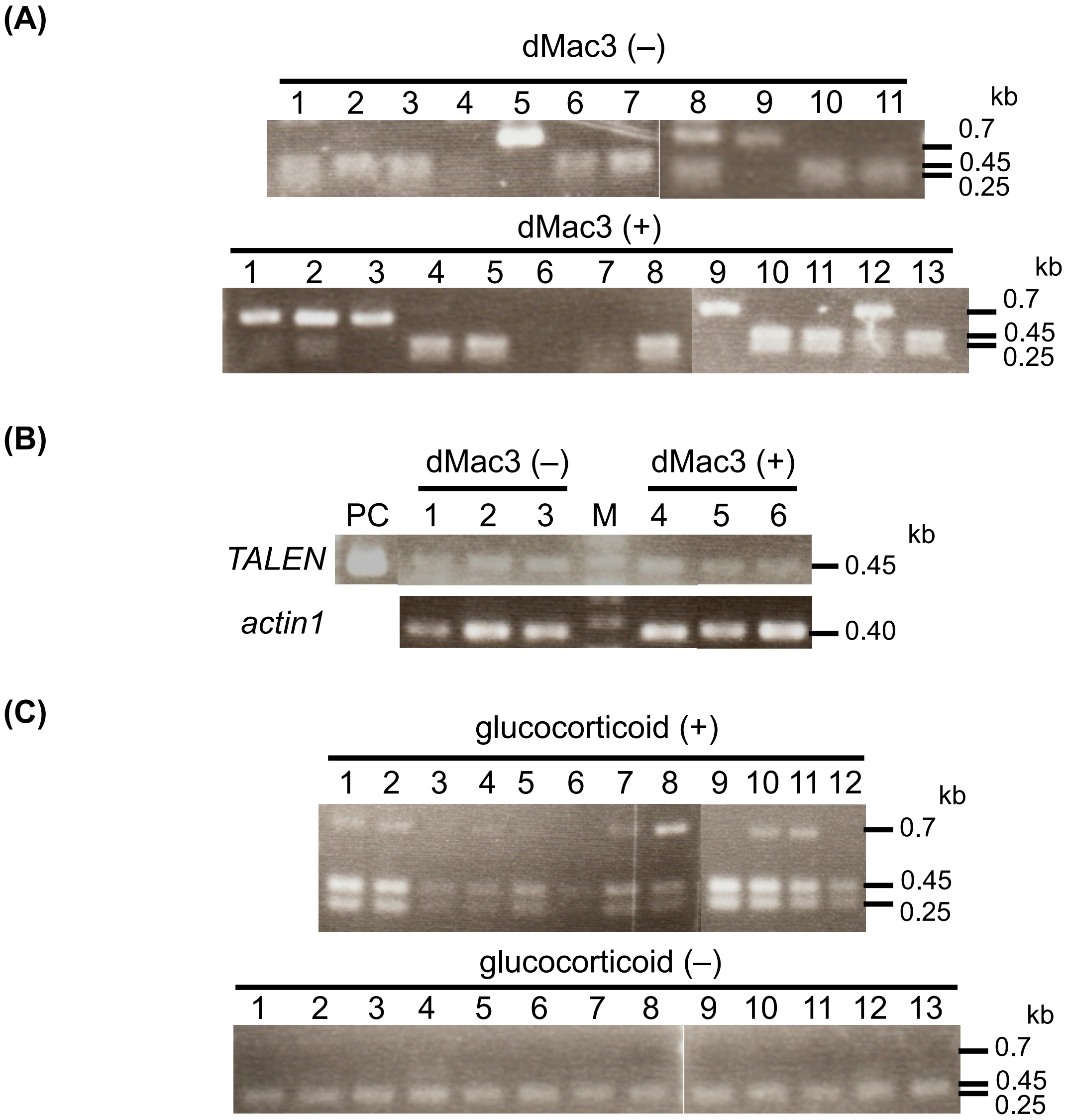

Fig B. Detection of targeted mutation.
